# *Labisia pumila* Prevents Complications of Osteoporosis by Increasing Bone Strength in a Rat Model of Postmenopausal Osteoporosis

**DOI:** 10.1155/2012/948080

**Published:** 2012-09-09

**Authors:** Siti Noor Fathilah, Shahrum Abdullah, Norazlina Mohamed, Ahmad Nazrun Shuid

**Affiliations:** ^1^Department of Pharmacology, Universiti Kebangsaan Malaysia Medical Center, Jalan Raja Muda Abdul Aziz, 50300 Kuala Lumpur, Malaysia; ^2^Division of Pharmacology, Department of Human Anatomy, Faculty of Medicine and Health Sciences, Universiti Putra Malaysia, 43400 Serdang, Selangor, Malaysia; ^3^Department of Mechanical and Materials Engineering, Faculty of Engineering and Built Environment, The National University of Malaysia (UKM), 43600 Bangi, Selangor, Malaysia

## Abstract

Estrogen replacement therapy (ERT) is the main treatment postmenopausal osteoporosis. However, ERT causes serious side effects, such as cancers and thromboembolic problems. *Labisia pumila var. alata* (LPva) is a herb with potential as an alternative to ERT to prevent complications of osteoporosis, especially fragility fractures. This study was conducted to determine the effects of LPva on the biomechanical strength of femora exposed to osteoporosis due to estrogen deficiency, using the postmenopausal rat model. Thirty-two female rats were randomly divided into four groups: Sham-operated (Sham), ovariectomized control (OVXC), ovariectomized with *Labisia pumila var. alata* (LP), and ovariectomized with ERT (Premarin) (ERT). The LPva and ERT were administered via oral gavage daily at doses of 17.5 mg/kg and 64.5 **μ**g/kg, respectively. Following two months of treatment, the rats were euthanized, and their right femora were prepared for bone biomechanical testing. The results showed that ovariectomy compromised the femoral strength, while LPva supplementation to the ovariectomized rats improved the femoral strength. Therefore, LPva may be as effective as ERT in preventing fractures due to estrogen-deficient osteoporosis.

## 1. Introduction

Osteoporosis is defined as a systemic skeletal disease that is characterized by low bone mass and microarchitectural deterioration of bone tissue, with a consequent increase in bone fragility and susceptibility to fracture [1]. According to the World Health Organization [2], osteoporosis occurs when the bone mineral density falls more than 2.5 standard deviations (SD) below the standard reference for maximum bone mineral density of young adult females. After the age of 35 to 40, the bone mass in females begins to decline slowly, but the rate of bone loss increases dramatically after menopause or ovariectomy due to estrogen deficiency.

By the age of 50, the bone mass in women is only two-thirds of that in men [3]. The combination of lower initial adult bone mass and faster rate of bone loss produce a higher incidence of osteoporosis in elderly women compared with men [4]. About one in three women aged more than 50 experienced an osteoporotic fracture in their lifetime [5].

Estrogen replacement therapy (ERT) is the main form of treatment and prevention of postmenopausal osteoporosis. Estrogen given alone or in combination with progesterone is able to prevent postmenopausal osteoporosis effectively [6]. Estrogen binds to estrogen receptors on the osteoclast surface, which causes the release of chemical mediators and reduction of osteoclastic activity, and therefore inhibits bone resorption [7].

The Women's Health Initiative study found that women who took ERT have slightly higher rates of breast cancer, ovarian cancer, heart attack, stroke, thromboembolism, and Alzheimer's disease [8–10]. Due to the numerous side effects of ERT, alternative antiosteoporotic agents that are comparable in effectiveness to estrogen but with minimal side effect are being investigated. These include soy [11], blueberry [12], and *Achyranthes bidentata* [13]. A histomorphometric study by Fathilah et al. (2012) [14] found that *Labisia pumila var. alata* has potential as an alternative to ERT for the prevention of postmenopausal osteoporosis. In a different study by Nazrun et al. (2010) [15], *Labisia pumila var. alata* was found to produce beneficial effects similar to estrogen on bone biomarkers in the postmenopausal osteoporosis animal model.

*Labisia pumila *(LP), a herbal plant from the family of* Myrsinaceae,* is a popular herb among women folk in Malaysia and is known locally as “Kacip Fatimah.” There are three types of *Labisia pumila*: *Labisia pumila var. alata* (LPva), *Labisia pumila var. pumila* (LPvp), and *Labisia pumila var. lanceolata* (LPvl) [16]. Traditionally, *Labisia pumila* extract is prepared by boiling the roots, leaves, or the whole plant in water, whereby the extract is then taken orally [17, 18]. It is used to facilitate labour, shrink the uterus, and improve menstrual irregularities and as postpartum medicine [17, 19]. Its exclusive use in women has led to the belief that it is a phytoestrogen, a compound with similar chemical structure to estrogen [20]. Therefore it is able to relieve menopausal symptoms. Several studies have demonstrated the estrogenic properties of LPva. It was found to inhibit estradiol binding to antibodies against estradiol [21], increase the uterine weight of ovariectomized rats [22], exert a specific estrogenic effect on human endometrial adenocarcinoma cells (Ishikawa-Var I line) [23], and initiate lipolysis in adipose tissue in a manner similar to estrogen [24]. In ovariectomized rats, LPva was also found to downregulate 11-*β* hydroxysteroid dehydrogenase1expression in adipose and liver tissues and decrease serum corticosterone levels [25].

Based on the possible estrogenic activities of LPva, it may be a suitable alternative to replace estrogen for the treatment and prevention of postmenopausal osteoporosis. Thus, it may also be effective in preventing complications of osteoporosis, especially fractures, by decreasing bone fragility.

Bone strength is the best and true indicator of bone function. However, it can only be directly assessed in animal models because the bone has to be tested until it fractures. In humans, it can only be indirectly assessed by using computer softwares [26]. The bone biomechanical test is the best method to measure bone strength directly, but it requires exerting a load to the bone until it fractures, which is impossible to be conducted in humans. The strength and stiffness of a bone are important parameters to determine its ability to resist fracture. Thus, improvement in these parameters will be beneficial in preventing fragility fractures [27]. A previous study demonstrated that supplementation of vitamin E, especially GTT, can improve bone structural and biomechanical properties of normal male rats [28]. The present study aimed to study in detail the effects of LPva on bone biomechanical strength in ovariectomized rats.

## 2. Materials and Methods

### 2.1. Animal and Treatment

Thirty-two female *Wistar* rats, with the average age of three months and weighing between 200 to 250 g, were used in this study. The rats were allowed to acclimatize for a week before being used for the study. The rats were housed two per cage, at normal room temperature with adequate ventilation and normal 12-hour light-dark cycle. All rats were allowed free access to water and food (commercial laboratory rat's food containing 0.97% calcium, 0.85% phosphorus, and 1.05 IU/g of Vitamin D3) (Gold Coin, Selangor, Malaysia). They were equally divided into four main groups. The sham-operated group (Sham) and the ovariectomized control group (OVXC) were given oral gavages of deionized water (vehicle). The treatment groups were given *Labisia pumila var. alata *at 17.5 mg/kg/day (LPva) and Premarin at 64.5 *μ*g/kg/day (ERT) daily for 8 weeks via oral gavages. The ERT group acted as positive control. After 8 weeks of treatments, the rats were euthanised. The right femora were dissected out and cleaned of any tissues. The distal femora were divided sagittally into two halves and wrapped with gauze dipped in phosphate-buffered saline. The approval for this study was obtained from the University Animal Ethic Committee of Universiti Kebangsaan Malaysia (PP/FAR/2009/NAZRUN/14 JULY/267-JULY 2009-MAY-2010).

### 2.2. *Labisia pumila var. alata* (LP) Extract

The LPva extract was supplied by Phytes Biotek Sdn Bhd. (Malaysia), a Good Manufacturing Practice (GMP) licensed manufacturer of herbal products, in the form of a freeze-dried standardized extract (Batch no: KF071107). The extraction was done at a factory in Shah Alam, Selangor, Malaysia, using a patented high-pressure water extraction process (US 7,132,117 B2), filtered at 1–4 mm and freeze-dried without maltodextrin or lactose. The extract was obtained from the root of the LPva plant and was the same extract that had been used previously by Fathilah et al., 2012 [14] and Nazrun et al., 2010 [15]. This extract was also the same form used for human consumption as health supplements. The extract was sent to the Forest Research Institute Malaysia (FRIM) for phytochemical testing. Based on the phytochemical test, the LPva extract that was used in this study contained flavonoids, saponins, and tritepenes.

The brownish powdered extract was dissolved in deionised water and given to the LPva treatment group via oral gavage at the dose of 17.5 mg/kg rat weight daily at 9 am for 8 weeks [14, 15]. The Premarin (Wyeth-Ayerst, Canada) tablet containing 0.625 mg of conjugated estrogen was crushed, dissolved in deionised water, and given to the ERT group via oral gavages at the dose of 64.5 *μ*g/kg rat weight daily at 9 am for 8 weeks [14, 15]. These doses were chosen based on our previous studies, which have demonstrated that LPva has the potential to be used as an alternative to ERT for the prevention of postmenopausal osteoporosis [5, 14]. In order to reduce the number of rats used in this study, we have followed the recommendation by the Animal Ethics Committee to use only one dose of LPva and Premarin, respectively.

### 2.3. Bone Biomechanical Test

Each right femur was wrapped with gauze dipped in phosphate-buffered saline, rewrapped with aluminum foil, and tested within two hours after dissection. Samples were kept moist at all times during the preparation procedure. The biomechanical properties of the femoral bones were assessed using an Instron Universal Testing Machine (model 5848; Microtester; Instron, Canton, MA, USA) that was equipped with Bluehill 2 software. Each femur was placed in a three-point bending configuration, whereby it was placed on two lower supports that were 5 mm apart. Force was applied at the middiaphysis on the anterior surface of the bone, causing the anterior surface to be in compression and the posterior surface in tension until it fractured. The load, stress, and strain parameters were recorded by the software. Graphs of stress against strain were also plotted. The slope value of the stress-strain curve in the elastic deformation region represents the modulus of elasticity (Young's modulus) of the femur. The main parameters of the bone mechanical test may be divided into extrinsic and intrinsic parameters; the extrinsic parameters (load, displacement, and stiffness) measure the properties of the whole bone, whereas the intrinsic parameters (stress, strain, and modulus of elasticity) measure the material of the bone.

### 2.4. Statistical Analysis

The results were expressed as mean ± standard error of the mean (SEM). The data analysis was performed using the Statistical Package for Social Sciences software (SPSS 17; SPSS, Chicago, IL, USA). The data were tested for normality using the Kolmogorov-Smirnov test. For normally distributed data, the statistical tests used were the analysis of variance (ANOVA), followed by Tukey's Honestly Significant Difference (HSD) test. For data that were not normally distributed, Kruskal-Wallis and Mann-Whitney tests were used.

## 3. Results

**Figure 1:**
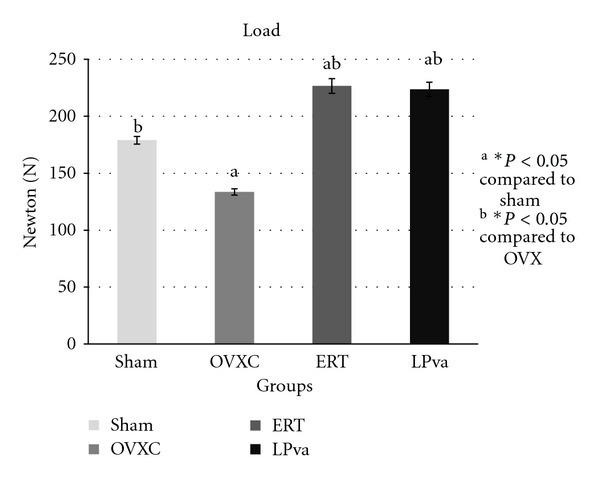
Load values: the load parameter measured the force received by the femur before it fractured. Sham (water vehicle), OVXC (water vehicle), LPva (*Labisia pumila var. alata *17.5 mg/kg/day), and ERT (Premarin 64.5 *μ*g/kg/day). Value expressed as mean ± SEM; *P* < 0.05 is considered significant. ^a^Significantly different from Sham group; ^b^significantly different from OVXC group.

**Figure 2:**
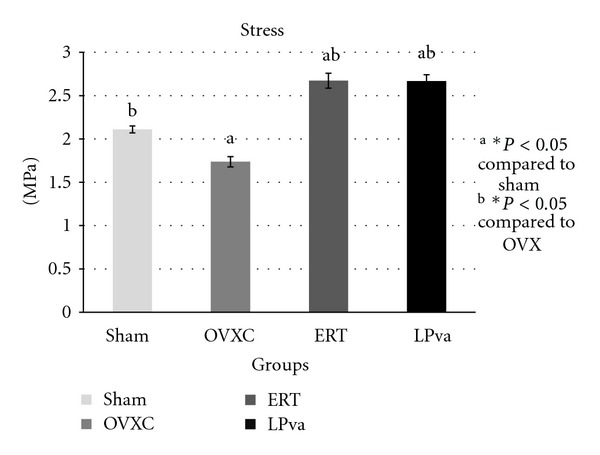
Stress values: the stress parameter measured the load per unit area received by the femur before it fractured. Sham (water vehicle), OVXC (water vehicle), LPva (*Labisia pumila var. alata *17.5 mg/kg/day), and ERT (Premarin 64.5 *μ*g/kg/day). Value expressed as mean ± SEM; *P* < 0.05 is considered significant. ^a^Significantly different from Sham group; ^b^significantly different from OVXC group.

**Figure 3:**
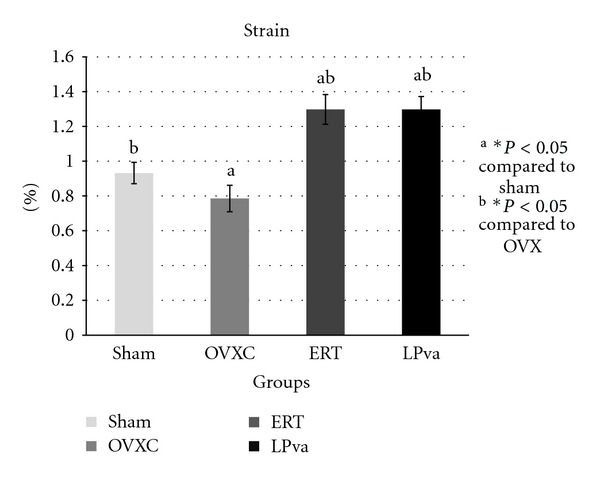
Strain values: the strain parameter measured the relative deformation of the femur caused by the stress before it fractured. Sham (water vehicle), OVXC (water vehicle), LPva (*Labisia pumila var. alata *17.5 mg/kg/day), and ERT (Premarin 64.5 *μ*g/kg/day). Value expressed as mean ± SEM; *P* < 0.05 is considered significant. ^a^Significantly different from Sham group; ^b^significantly different from OVXC group.

**Figure 4:**
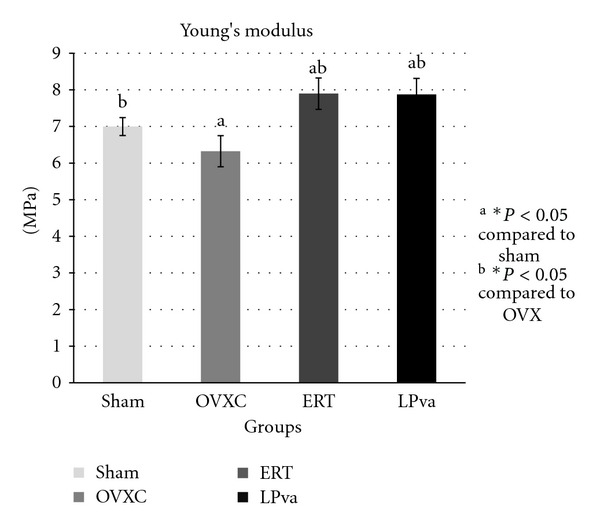
Young's modulus values: the modulus of elasticity (Young's modulus) measured the tendency of the femur to be deformed elastically when a force was applied to it. Sham (water vehicle), OVXC (water vehicle), LPva (*Labisia pumila var. alata *17.5 mg/kg/day), and ERT (Premarin 64.5 *μ*g/kg/day). Value expressed as mean ± SEM. *P* < 0.05 is considered significant. ^a^Significantly different from Sham group; ^b^significantly different from OVXC group.

Femoral strength was evaluated using biomechanical tests (Figures 1–4). The load parameter measured the force received by the femur before it fractured (Figure 1). The ERT group received significantly greater load compared to the Sham and OVXC groups. The LPva group received comparable load to ERT and significantly greater load as compared to OVXC group.

The stress parameter measured the load per unit area received by the femur before it fractured (Figure 2). The ERT group received significantly higher stress than the Sham and OVXC groups. The LPva group received higher stress than the OVXC group and was comparable to the ERT group. The OVXC group received the lowest stress compared to other groups. The strain parameter measured the relative deformation of the femur caused by the stress before it fractured (Figure 3). The ERT and LPva groups had significantly higher strain than the Sham and OVXC groups. The OVXC group had the lowest strain compared to other groups. The modulus of elasticity (Young's modulus) measured the tendency of the femur to be deformed elastically when force is applied to it (Figure 4). The ERT and LPva groups had a significantly higher modulus of elasticity compared to other groups. The OVXC group had the lowest modulus of elasticity compared to the other groups.

## 4. Discussion

Hormone or estrogen replacement therapy (HRT/ERT) has been used for the prevention and treatment of postmenopausal osteoporosis, but it may cause serious side-effects (Ferguson, 2004) [29]. It was reported that women who took HRT have slightly higher rates of breast cancer, ovarian cancer, heart attack, stroke, thromboembolism, and Alzheimer's disease [8–10]. Due to the numerous side effects of ERT, we have investigated the potential of LPva as an alternative treatment for postmenopausal osteoporosis in terms of enhancing the bone resistance to fracture. This herbal plant was selected due to its phytoestrogenic properties [20, 21]. LPva has been found to protect the bone of estrogen-deficient rat in a histomorphometric study [14].

To the best of our knowledge, this is the first report on the effects of LPva on the bone biomechanical strength in an ovariectomized rat model. The effects of LPva on the biomechanical parameters of postmenopausal osteoporosis rat model were compared to ERT, the gold standard treatment for postmenopausal osteoporosis. Rats have become a widely accepted model of human bone disease because their mechanism of controlling the gain and loss of bone mass are similar to humans. An increase in bone mass was observed in longitudinal bone growth and modelling drifts with bone loss related to bone remodelling. Furthermore, the response to mechanical influences, hormones, drugs, and other agents in rats are similar to humans [30]. Young adult rats were selected as the animal model in this study for their dynamic bone growth, which represents the critical bone growth phase of the young adult humans in their twenties. This phase requires an optimum bone growth to achieve the peak bone mass. During this phase, more bones are formed than resorbed in each remodelling cycle. If the peak bone mass is not fully optimized or is disturbed by factors such as unstable hormones, the risk of developing osteoporosis in the elderly years is higher [31].

Similar doses of 17.5 mg/kg of LPva and 0.0645 mg/kg of ERT were used in the present study as those that were used in the study by Fathilah et al. (2012) [14], which found that these doses were effective in the prevention of osteoporosis in the ovariectomised rat model. It was shown that the supplementation of 17.5 mg/kg of LPva to ovariectomized rats for 8 weeks was able to prevent osteoporotic changes that were reflected in the bone biochemical markers [15]. In terms of safety, the LPva extract was found to exhibit no-adverse-effect level (NOAEL) at the dose of 50 mg/kg in a subacute toxicity study [32], 1000 mg/kg in a subchronic toxicity study [33], and 800 mg/kg in a reproductive toxicity study [34]. Fazliana et al. (2009) [22] had used several doses of LPva ranging from 10 to 50 mg/kg, but found that only the 50 mg/kg dose was able to suppress weight gain in ovariectomized rats. The dose of estrogen used in the same study was 0.625 mg/kg, which was higher than the estrogen dose used in our study. However, our lower estrogen dose was effective as this dose was able to prevent bone changes induced by ovariectomy as evidenced by the improvement seen in all the bone biomechanical parameters. In fact, these bone biomechanical parameters were significantly better than the sham group.

The bone loss associated with estrogen deficiency is generally attributed to increased bone resorption and increased bone turnover. There was evidence to suggest that estrogen may exert anabolic effects on bone. Estrogen has been shown to stimulate the differentiation and activity of osteoblasts [35, 36] and increase bone formation and bone mass in animal models [37, 38]. More interestingly, the LPva group also demonstrated this anabolic effect similar to ERT. The structural improvement by ERT and LPva should lead to stronger bones as bone structure determines their strength. Since no study has been done on the effect of LPva on bone strength, we have carried out bone biomechanical testing and found that ERT and LPva supplementation significantly improved both extrinsic parameters (load) and intrinsic parameters. The findings of the bone biomechanical test suggested that ERT and LPva enhanced the bone biomechanical properties of ovariectomized rats.

A recent study by Fathilah et al. (2012) [14] on bone histomorphometric analysis demonstrated that supplementation of LPva in ovariectomized rats was as effective as ERT in preventing osteoporotic changes. Based on previous studies, there are several possible mechanisms of LPva in protecting the bone against estrogen deficiency. The most likely mechanism is due to its phytoestrogenic actions [23, 39]. LPva contains triterpene saponins, including the compound ardisiacrispin A, which were thought to interact with estrogen receptors [40].

LPva was also found to have similar antioxidative properties as those exhibited by beta carotene, flavonoids, vitamin C, total anthocyanins, and phenolics [41]. LPva extract demonstrated a potent antioxidant activity comparable to that of ascorbic acid, one of the strongest known antioxidants [42]. The other possible mechanism of action of LPva against osteoporosis is via its antioxidative properties as demonstrated by tocotrienols, another potent antioxidant [28]. The antioxidant activity of LPva is contributed by its flavanoids, ascorbic acid, beta carotene, anthocyanin, and phenolic compounds [43]. Tumour necrosis factor-*α* (TNF-*α*) is a bone-resorbing cytokine that promotes bone resorption by activating mature osteoclasts or by stimulating proliferation and differentiation of osteoclasts [44]. Inhibition of this cytokine may be another possible mechanism of action that LPva exhibits against osteoporosis. It has been shown that blocking the effect of TNF-*α* prevented postovariectomy bone loss [45]. LPva was found to suppress the TNF-*α* level to below the baseline level in cultured HaCat cells [46].

As a conclusion, based on the comparable effects of LPva to ERT on bone biomechanical testing and its safety profile, LPva has the potential to prevent osteoporotic fractures in the postmenopausal or estrogen-deficient state. It may be taken as supplements by postmenopausal women who are afraid of the side effects of estrogen [46]. LPva seemed to be safer than Premarin as it exhibited no reproductive toxicity in animal at forty-five times higher than the dose used in the present study [33]. Further studies are required to determine its antiosteoporotic mechanism of action for the prevention of complications of osteoporosis.
